# Saccharide Substituted Zinc Phthalocyanines: Optical Properties, Interaction with Bovine Serum Albumin and Near Infrared Fluorescence Imaging for Sentinel Lymph Nodes

**DOI:** 10.3390/molecules19010525

**Published:** 2014-01-03

**Authors:** Li Lu, Feng Lv, Bo Cao, Xujun He, Tianjun Liu

**Affiliations:** Tianjin Key Laboratory of Biomedical Materials, Institute of Biomedical Engineering, Chinese Academy of Medical Sciences & Peking Union Medical College, Tianjin 300192, China

**Keywords:** near infrared fluorescence probe, sentinel lymph node imaging, zinc phthalocyanine, saccharide, bovine serum albumin

## Abstract

Saccharide-substituted zinc phthalocyanines, [2,9(10),16(17),23(24)-tetrakis((1-(β-d-glucose-2-yl)-1*H*-1,2,3-triazol-4-yl)methoxy)phthalocyaninato]zinc(II) and [2,9(10),16(17),23(24)-tetrakis((1-(β-d-lactose-2-yl)-1*H*-1,2,3-triazol-4-yl)methoxy)phthalocyaninato]zinc(II), were evaluated as novel near infrared fluorescence agents. Their interaction with bovine serum albumin was investigated by fluorescence and circular dichroism spectroscopy and isothermal titration calorimetry. Near infrared imaging for sentinel lymph nodes *in vivo* was performed using nude mice as models. Results show that saccharide- substituted zinc phthalocyanines have favourable water solubility, good optical stability and high emission ability in the near infrared region. The interaction of lactose-substituted phthalocyanine with bovine serum albumin displays obvious differences to that of glucose- substituted phthalocyanine. Moreover, lactose-substituted phthalocyanine possesses obvious imaging effects for sentinel lymph nodes *in vivo*.

## 1. Introduction

Optical imaging has wide applications in biomedical diagnosis due to its low cost, low-energy radiation, high sensitivity and non-invasive nature [1,2]. With the development of imaging technology, near infrared (NIR) fluorescence probes show great potential for non-invasive *in vivo* imaging owing to their low tissue autofluorescence and deep tissue penetration [3,4]. They have generated considerable research interest in several areas of the biomedical field, including vascular mapping, tissue perfusion, inflammation monitoring, tumor diagnosis, sentinel lymph node (SLN) imaging, and so on [5,6,7,8]. Especially, some fluorescent probes have been translated to the clinic for SLN imaging [9,10]. Indocyanine green (ICG) is an approved NIR fluorescence probe for clinical use, but its low quantum efficiency and rapid metabolism limits the imaging sensitivity [11]. Other organic dyes or inorganic quantum dots have also been reported for SLN imaging in animal tests or clinical applications [12,13,14], however they still far from being the ideal fluorescence imaging probe. Thus, it is important to develop new high performance NIR fluorescence probes for SLN imaging.

As a kind of fluorescence agent with large visible to NIR absorption and acceptable fluorescent efficiency, zinc phthalocyanines have been widely applied in several research fields, including materials science, photochemistry and biomedical science [15,16,17]. However, low water solubility and weak cell specificity limit their biomedical applications. By coupling with saccharide moieties, the water solubility, biocompatibility and cell specificity of zinc phthalocyanines can be improved [18,19]. Saccharide-substituted zinc phthalocyanines have been developed as photodynamic therapy (PDT) sensitizers or imaging probes [20,21,22,23,24]. Recently, several saccharide-substituted zinc phthalocyanines were developed for *in vivo* tumor imaging [22,23,24]. In particular, the saccharide-substituted zinc phthalocyanines incorporating heterocyclic triazoles moieties such as [2,9(10),16(17),23(24)-tetrakis((1-(β-d-glucose-2-yl)-1*H*-1,2,3-triazol-4-yl)methoxy)phthalocyaninato]zinc(II) and [2,9(10), 16(17),23(24)-tetrakis((1-(β-d-lactose-2-yl)-1*H*-1,2,3-triazol-4-yl)methoxy)phthalocyaninato]zinc(II), made using click chemistry, are attracting our attention as imaging probes [23,24]. Based on their favorable water solubility, good optical stability, high emission ability and excellent biocompability, saccharide-substituted zinc phthalocyanines can be expanded to SLN imaging as fluorescence probes.

As a diagnostic agent a fluorescence probe performs a pharmacological process just as same as any therapeutic drug. The pharmacological behavior of a fluorescence probe plays a critical role in determining its fate in the blood stream. The binding ability of fluorescence probes to proteins in the blood stream may have a significant impact on its distribution, availability and metabolism. Since albumins are the most abundant serum proteins in the circulatory system of a variety of organisms and can transport reversibly-bound drugs to their destination via drug-protein complex formation, they tend to be studied as a model serum protein for the assessment of drug-protein interactions [25,26]. Bovine serum albumin (BSA) is generally chosen to understand the drug-protein interaction as a convenient model because of its high sequence identity to that of human serum albumin [27,28]. However, although the interaction of some therapeutic drugs with protein has partly been studied and elucidated [29,30], there is few reports about diagnostic drugs. As a process at the molecular level of a small molecule and macromolecule, the interaction of a fluorescence probe with proteins is of critical importance and necessity.

In this paper, the study of saccharide-substituted zinc phthalocyanines, [2,9(10),16(17),23(24)-tetrakis((1-(β-d-glucose-2-yl)-1*H*-1,2,3-triazol-4-yl)methoxy)phthalocyaninato]zinc(II) and [2,9(10), 16(17),23(24)-tetrakis((1-(β-d-glucose-2-yl)-1*H*-1,2,3-triazol-4-yl)methoxy)phthalocyaninato]zinc(II), were expanded to SLN imaging besides their roles in tumor diagnosis. Moreover, the interaction of saccharide-substituted zinc phthalocyanines with proteins was investigated to explore the interaction process of fluorescence probes and proteins at the molecular level, which could pave the way for the design of improved fluorescence diagnostic agents.

## 2. Results and Discussion

**Figure 1:**
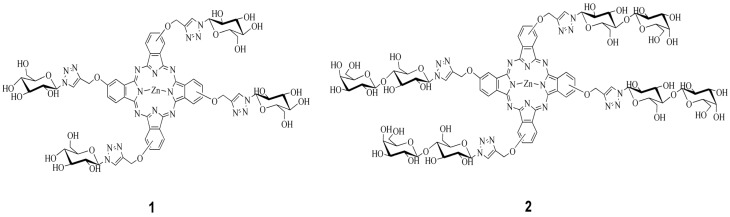
Structure of glucose-substituted zinc phthalocyanine (**1**) and lactose-substituted zinc phthalocyanine (**2**).

Saccharide-substituted zinc phthalocyanines,2,9(10),16(17),23(24)-tetrakis((1-(β-d-glucose-2-yl)-1*H*-1,2,3-triazol-4-yl)methoxy)phthalocyaninato]zinc(II) and 2,9(10),16(17),23(24)-tetrakis((1-(β-d-lactose-2-yl)-1*H*-1,2,3-triazol-4-yl)methoxy)phthalocyaninato]zinc(II) (Figure 1) were synthesized as green solids by condensation of their precursor saccharide-substituted phthalonitriles [23,24].

**Figure 2:**
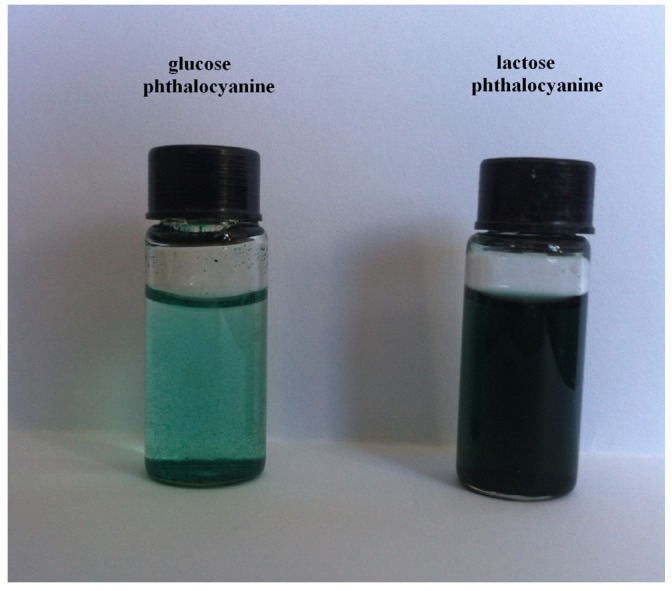
Saccharide-substituted zinc phthalocyanines in water solution at the concentration of 200 µM.

Traditionally phthalocyanine-carbohydrate conjugates were prepared through three step reactions including nucleophilic reaction of a carbohydrate with 4-nitrophthalonitrile, cyclotetramerisation reactions and carbohydrate deprotection [18,19]. Here, saccharide-substituted phthalonitriles were achieved via click reactions instead of traditional substitution reactions. The click reaction is particularly suitable for bioconjugations due to the tolerance of biological conditions and most polar functional groups with simple, easy workup procedures for the resulting products [31,32]. The water-solubility of saccharide-substituted zinc phthalocyanines was evaluated by their octanol/water partition coefficients. The octanol/water partition coefficients of glucose-substituted phthalocyanine and lactose-substituted phthalocyanine are 1.25 ± 0.03 and 0.94 ± 0.02, respectively. Obviously the better water solubility of lactose-substituted phthalocyanine comes from the presence of more hydrophilic hydroxyl groups in the saccharide unit. In addition, their water solubility was further proved by the appearance of their water solutions (Figure 2).

**Figure 3:**
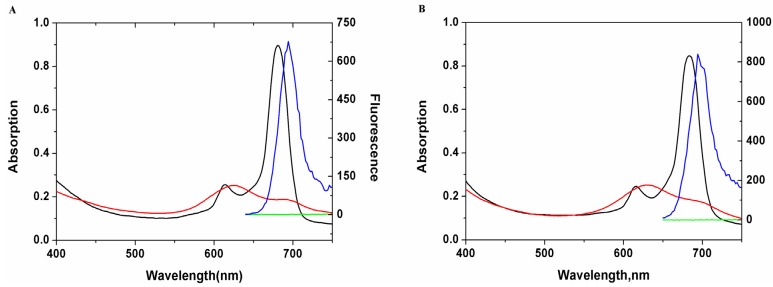
Absorption spectrum and fluorescence spectrum of saccharide substituted zinc phthalocyanines in DMSO (Abs: in black; FL: in blue) or water solution. (Abs: in red, FL: in green) (**A**) glucose substituted zinc phthalocyanine; (**B**) lactose substituted zinc phthalocyanine.

The absorption and fluorescence spectra of saccharide substituted zinc phthalocyanines in DMSO or in water were obviously different as shown in Figure 3. In the UV-Vis spectra, two typical Q-band absorptions at 618 nm and 682 nm in the NIR area indicate the non-aggregated states of saccharide- substituted zinc phthalocyanines in DMSO solution. However, when measured in water solution, there is only a weak and wide peak at 600–700 nm, which can be attributed to cofacial aggregation of saccharide-substituted phthalocyanines in water. Strong fluorescence signals of two saccharide- substituted phthalocyanines are detected at 690 nm with excitation wavelength at 618 nm in DMSO, while weak fluorescence signals are obtained in water solution under the same conditions. Interestingly, saccharide-substituted zinc phthalocyanines can be disaggregated in biotissues and display ideal fluorescence signals *in vivo* [23,24].

**Figure 4:**
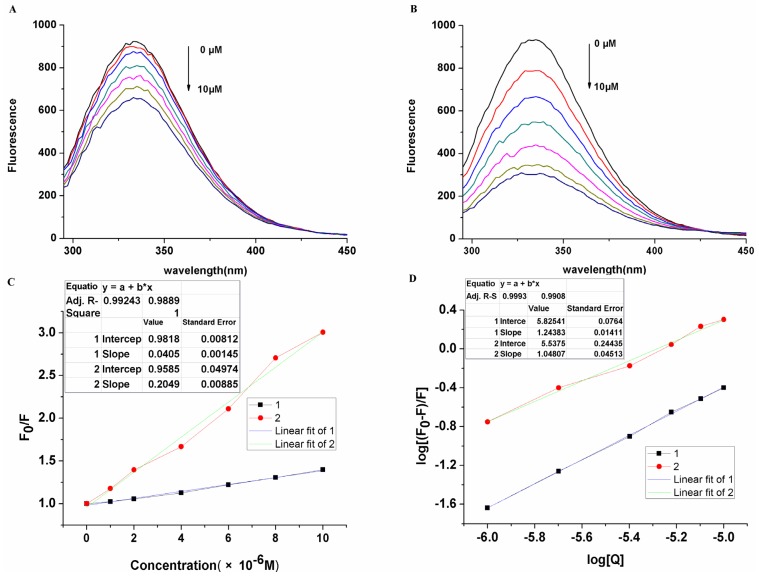
Fluorescence emission spectral changes of BSA (30 µM) on the addition of varying concentrations of saccharide substituted zinc phthalocyanines at the concentration of 0, 1, 2, 4, 6, 8, 10 µM. (**A**) glucose substituted zinc phthalocyanine; (**B**) lactose substituted zinc phthalocyanine; (**C**) linear relationship of fluorescence quenching; (**D**) logarithm relationship of fluorescence quenching.

The interaction of drugs with plasma proteins is of considerable pharmacological importance because the effects on organs or tissues depend on their binding to plasma proteins. This can provide important information about the structural features that determine the effectiveness of drugs. Fluorescence quenching methods, CD spectral analysis and ITC measurements can analyze the interaction of saccharide-substituted zinc phthalocyanines with BSA qualitatively and quantitatively. Due to the tyrosine, tryptophan, and phenylalanine resides of BSA, fluorescence is displayed at 340 nm with the excitation wavelength at 280 nm. It can be seen that the addition of saccharide-substituted phthalocyanines results in a concentration dependent quenching of the intrinsic fluorescence of BSA (Figure 4).

There is remarkable change of the emission maximum with the addition of lactose-substituted zinc phthalocyanine while weak change is seen with glucose-substituted zinc phthalocyanine. The changes in BSA fluorescence intensity are related to the saccharide-substituted phthalocyanines by the Stern-Volmer relationship [33]. The Stern-Volmer quenching constant k_SV_ of glucose-substituted zinc phthalocyanine and lactose-substituted zinc phthalocyanine with BSA are 4.06 × 10^4^ and 2.05 × 10^5^, respectively, which comes from the differences of their molecular structures. For static quenching, the number of binding sites (N) of glucose-substituted zinc phthalocyanine and lactose- substituted zinc phthalocyanine on BSA in water are 1.24 and 1.05. The N values suggest that saccharide-substituted zinc phthalocyanines form 1:1 adducts with BSA. The k_SV_ and N are typical of metallated phthalocyanine-BSA interactions in aqueous solution, which are not affected by the conjugation of saccharide molecules. Furthermore, the approximate binding ability of glucose- and lactose-substituted zinc phthalocyanine with BSA were determined by their binding constants of 6.69 × 10^5^ and 3.45 × 10^5^ without remarkable differences.

**Figure 5:**
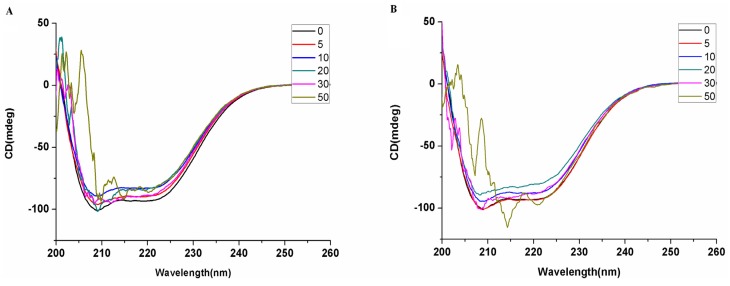
CD spectral changes of BSA (1 µM) on the addition of saccharide-substituted zinc phthalocyanines in water at the concentration of 0, 5, 10, 20, 30, 50 µM. (**A**) glucose-substituted zinc phthalocyanine; (**B**) lactose-substituted zinc phthalocyanine.

To get an insight into the structure of BSA, CD spectra were investigated for the interaction of saccharide-substituted zinc phthalocyanines with the BSA system (Figure 5). In the far ultraviolet region, the CD spectra of BSA exhibit two negative minima peaks of the α-helix structure at 208 and 217 nm. The interaction between glucose-substituted zinc phthalocyanine and BSA causes only a decrease in band intensity without any significant shift of the peaks at the lower ratio. When the ratio of glucose-substituted zinc phthalocyanine and BSA exceeds 20-fold, the CD spectra have an irregular change due to the structural damage of the protein, while in the lactose-substituted zinc phthalocyanine and BSA system under the same conditions, the protein structure is not damaged until the concentration of lactose-substituted zinc phthalocyanine is 30-fold than that of BSA. The results indicate that lactose-substituted zinc phthalocyanine induces a more slight decrease in the helix structure content of the protein.

**Figure 6:**
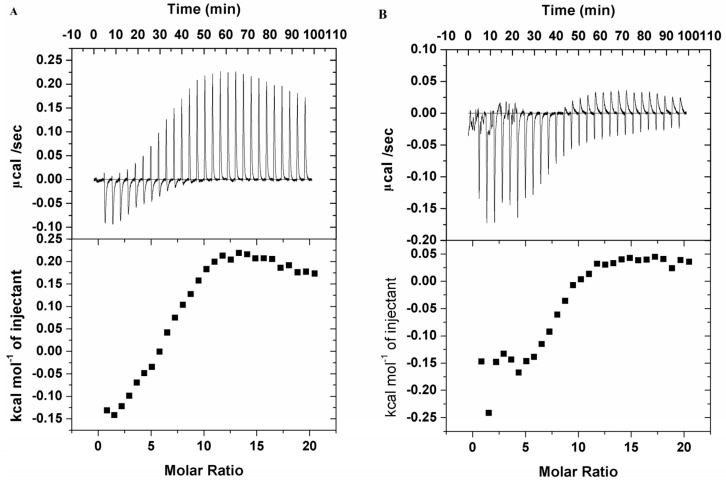
ITC data from the titration of 30 µM BSA in the presence of 300 µM saccharide-substituted zinc phthalocyanines. (**A**) glucose-substituted zinc phthalocyanine; (**B**) lactose-substituted zinc phthalocyanine.

In order to clarify the thermodynamic process, ITC was used to evaluate the binding interaction of saccharide-substituted phthalocyanines and BSA by quantifying the change in enthalpy entropy and Gibbs free energy as shown in Figure 6 at 25 °C. The thermodynamic processes of the two saccharide-substituted zinc phthalocyanines with BSA show obvious differences. The binding of lactose-substituted zinc phthalocyanine with BSA is exothermic in nature, with a negative enthalpy and positive entropy value. Using computational non-linear fitting analysis, the enthalpy change (∆H) of −260.3 ± 55.05 kcal mol^−1^ and entropy change (∆S) of 21.2 cal in the interaction process indicate that the binding reaction is enthalpically, as well as entropically driven. The negative enthalpy and positive entropy is consistent with the characteristic of weak van der Waals interaction and hydrophobic interaction during protein-ligand complex formation, respectively [34,35]. The negative free energy value suggests that the binding is an energetically feasible process giving a binding constant of 6.55 × 10^4^ and a number of binding sites of 5.34. However, the binding of glucose-substituted phthalocyanine with BSA is endothermic. The enthalpy change and entropy change cannot be calculated by the computational non-linear fitting analysis. This difference may be due to its aggregation from a homogeneous phase solution to a precipitation state with long time titration.

**Figure 7:**
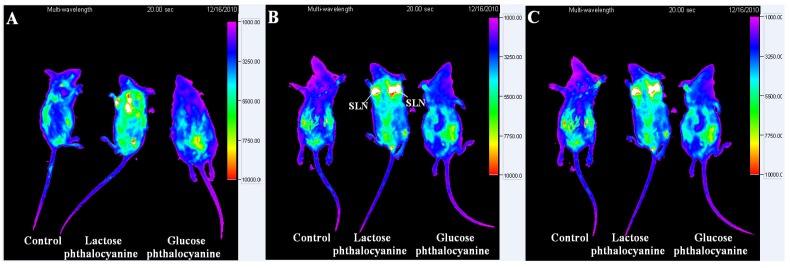
Optical imaging for SLN *in vivo* using saccharide-substituted zinc phthalocyanines as probes, with representative one of three in each group was given (injected with 100 µL of 200 µM with an exposure time of 20 s (filters: excitation 625 nm, emission 700 nm). (**A**) before administration; (**B**) after administration for 10 min; (**C**) after administration for 30 min.

**Figure 8:**
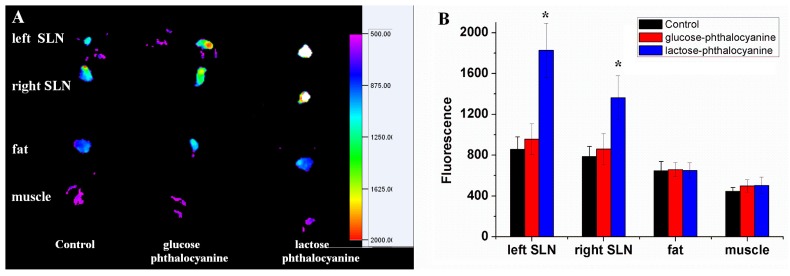
Distribution and fluorescence intensity of dissected organs. (**A**) *Ex vivo* imaging with an exposure time of 20 s (filters: excitation 625 nm, emission 700 nm); (**B**) Quantitative analysis of fluorescence intensity (*p* < 0.05, compared to control group, *n* = 3).

Whole animal imaging for SLN were performed in mice using the saccharide-substituted zinc phthalocyanines as fluorescence tags by subcutaneous injection in the left and right upper paws (Figure 7). The dosage of probe is chosen according to the use of ICG or Cy5.5 as optical imaging probes [11,36,37]. In those studies, the dosages of fluorescence probe were 1 mg/mL of 10–100 µL or 1–10 mg/kg, respectively. For saccharide-substituted zinc phthalocyanine, the dosage of 200 µL at the concentration of 200 µM (0.33 mg/mL or 0.45 mg/mL) is a rational choose in one case it is followed in the range of their dosages, in another case we found that high quality imaging and healthy animals can be achieved with this dosage of probe. Our formal experiment also demonstrated this dosage is suitable for *in vivo* imaging [23,24]. Here, in the lactose-substituted phthalocyanines group, strong fluorescence is observed in SLN after administration for 10 min, while little fluorescence is displayed in the glucose-substituted zinc phthalocyanine group. After permeation and metabolism for 30 min, there is still strong fluorescence in SLN of the lactose-substituted zinc phthalocyanine group. Lactose-substituted zinc phthalocyanine can remain in SLN for long time in view of its special binding to biotissue, whereas glucose-substituted phthalocyanine cannot show obvious imaging for SLN after the administration is performed for 30 min. The obvious difference of lactose- and glucose-substituted zinc phthalocyanines for SLN imaging is mainly related to their structures and properties. Next, the mice were sacrificed after 30 min and the SLN, muscle and fat tissues were harvested for *ex vivo* analysis of the probe biodistribution. Quantification of fluorescence intensity in tissues is shown in Figure 8. Consisted with the *in vivo* imaging, the SLN of lactose-substituted zinc phthalocyanine group has intense fluorescence signals while the muscle and fat tissues organs have only background fluorescence signals. In the glucose-substituted zinc phthalocyanine group, the weak fluorescence of SLN cannot be detected *in vivo* due to the absorption and peameation of the biotissues. Lactose-substituted zinc phthalocyanine is thus a more suitable fluorescence probe for SLN imaging than glucose-substituted zinc phthalocyanine. Besides, the toxicity studies of saccharide-substituted zinc phthalocyanines have been illustrated by histological analysis, which show that these saccharide-substituted zinc phthalocyanines at rational doses caused no damage to the organs according to the HE staining [23,24]. This proves the safety of these NIR fluorescence probes *in vivo*.

Nowadays the NIR imaging agents in clinical use are ICG and Methylene Blue, which have been received approval from the U.S. Food and Drug Administration (FDA) for clinical use. The excitation wavelength of ICG is 780 nm, while that of Methylene Blue is 660 nm. Although they offer many advantages for fluorescence imaging, their drawback, including complex synthesis, high price and low quantum efficiency, hamper their wide application. Other potential NIR imaging agentd such as cy5.5 and quantum dotd have the disadvantages of poor water solubility and high price. Compared with the above imaging agents, saccharide-substituted zinc phthalocyanines have simple preparation, low price, high quantum efficiency and favorable water solubility. More importantly, the high fluorescence imaging *in vivo* of saccharide-substituted zinc phthalocyanines suggest they have a bright future for clinical use.

## 3. Experimental

### 3.1. Materials

Glucose substituted zinc phthalocyanine [2,9(10),16(17),23(24)-tetrakis((1-(β-d-glucose-2-yl)-1*H*-1,2,3-triazol-4-yl)methoxy)phthalocyaninato]zinc(II) and lactose-substituted zinc phthalocyanine [2,9(10),16(17),23(24)-tetrakis((1-(β-d-lactose-2-yl)-1*H*-1,2,3-triazol-4-yl)methoxy) phthalocyanin-ato]zinc(II) were synthesized in our lab [23,24]. Bovine serum albumin whose average molar mass is 66.0 kg mol^−1^ was purchased from Beijing Dingguo Biotech Company (Beijing, China). All purchased materials were used without further purification.

### 3.2. Octanol/Water Partition Coefficients of Saccharide Substituted Zinc Phthalocyanines

Water-solubility of saccharide substituted zinc phthalocyanines was determined by measuring their octanol/water partition coefficents by reverse-phase thin-layer chromatography (TLC). Commercially available reverse-phase thin-layer plates were used as stationary phase and methanol/water (2:1 *v*/*v*) mixtures were preferably used as mobile phase. Octanol/water partition coefficients were calculated on the basis of traditional method of TLC with salicylaldehyde as a reference compound [38].

### 3.3. UV-Vis and Fluorescence Spectra of Saccharide Substituted Zinc Phthalocyanines

UV-Vis and fluorescence spectra were recorded on a ThermoFisher Scientific Varioskan™ Flash multimode microplate spectra photometer (Thermo Fisher Scientific Inc, Waltham, MA, USA). Using a small volume with 200 µL sample at the concentration of 20 μM in DMSO or water solution, an Expert 96 AsysHitec well plate reader (Thermo Fisher Scientific Inc, Waltham, MA, USA) was employed for absorption and fluorescence measurements.

### 3.4. Interaction of Saccharide Substituted Zinc Phthalocyanines with BSA

Interaction of saccharide-substituted zinc phthalocyanines with BSA was evaluated by fluorescence and circular dichroism spectroscopy and isothermal titration calorimetry. An aqueous solution of BSA (30 µM) was titrated with varying concentrations of the respective saccharide-substituted zinc phthalocyanines by fluorescence spectra. The fluorescence of BSA was recorded between 295 nm and 450 nm at the exciting wavelength of 280 nm. Then the alterations in the secondary structure of the protein in the presence of saccharide-substituted zinc phthalocyanines were studied on a Jasco J-810 spectropolarimeter (Jasco, Tokyo, Japan). Spectra were measured as the average of three scans from 250 to 200 nm at a scan rate of 200 nm/min. An aqueous solution of BSA (1 µM) was titrated with molar ratios of zinc phthalocyanines and BSA from 0 to 50. Finally, the interaction thermodynamics of saccharide substituted zinc phthalocyanines with BSA were measured using a VP-ITC calorimeter (GE Healthcare, Piscataway, NJ, USA). An aqueous solution of BSA (30 µM) was titrated with varying concentrations of the respective saccharide substituted zinc phthalocyanines. The data were analyzed to determine the binding stoichiometry (N), and affinity constant (K) using the Origin software (OriginLab Corporation, Wellesley Hills, MA, USA).

### 3.5. *In Vivo* Imaging and Distribution of Saccharide Substituted Zinc Phthalocyanines for SLN

Athymic nude mice (seven weeks old, 20–25 g) were used. All the animal experiments were performed in compliance with the Guiding Principles for the Care and Use of Laboratory Animals, Peking Union Medical College, China. Animals had free access to food and water. Athymic nude mice were randomly assigned to different groups as follows: glucose substituted zinc phthalocyanine group, lactose substituted zinc phthalocyanine group, control group (*n* = 3 for each group). In the experimental group, saccharide-substituted zinc phthalocyanine solution was administered by subcutaneous injection in the left or right upper paw at 100 µL of 200 µM. Fluorescence images *in vivo* were taken using a Kodak Image Station *in vivo* FX (CareStream, Rochester, NY, USA*)* (filters: excitation 625 nm, emission 700 nm) with an exposure time of 20 s after administration for 10 min and 30 min respectively. At the end of the imaging, anesthetized mice were sacrificed and images of SLN, fat tissue and muscle tissue were made to evaluate the distribution of near infrared fluorescence agents. Fluorescence images of tissues were analyzed using the Kodak Image Analysis Software.

### 3.6. Statistical Analysis

The statistical analysis was performed by Bonferroni *t*-test for comparison with the control group. The values were presented as the means ± SD of three tests and statistical significance was determined at *p* < 0.05.

## 4. Conclusions

In summary, the uses of the saccharide-substituted phthalocyanines, [2,9(10),16(17),23(24)-tetrakis((1-(β-d-glucose-2-yl)-1*H*-1,2,3-triazol-4-yl)methoxy)phthalocyaninato]zinc(II) and [2,9(10), 16(17),23(24)-tetrakis((1-(β-d-lactose-2-yl)-1*H*-1,2,3-triazol-4-yl)methoxl)phthalocyaninato]zinc(II), were expanded to SLN imaging besides their roles in tumor diagnosis. Saccharide-substituted zinc phthalocyanines have favourable water solubility, good optical stability and high emission ability in the near infrared region. The interaction of lactose-substituted phthalocyanine with bovine serum albumin displays obvious differences to that of glucose-substituted phthalocyanine. Their interaction with proteins may be used to explore the molecular processes of interaction between fluorescence probes and proteins, which would pave the way for the design of improved fluorescence diagnostic agents.

## References

[1] Weissleder R., Pittet M.J. (2008). Imaging in the era of molecular oncology. Nature.

[2] Luker G.D., Luker K.E. (2008). Optical imaging: Current applications and future directions. J. Nuclear Med..

[3] Hilderbrand S.A., Weissleder R. (2010). Near-infrared fluorescence: Application to *in vivo* molecular imaging. Curr. Opin. Chem. Biol..

[4] Kobayashi H., Ogawa M., Alford R., Choyke P.L., Urano Y. (2010). New strategies for fluorescent probe design in medical diagnostic imaging. Chem. Rev..

[5] Rao J., Dragulescu-Andrasi A., Yao H. (2007). Fluorescence imaging *in vivo*: Recent advances. Curr. Opin. Biotechnol..

[6] Magalotti S., Gustafson T.P., Cao Q., Abendschein D.R., Pierce R.A., Berezin M.Y., Akers W.J. (2013). Evaluation of inflammatory response to acute ischemia using near-infrared fluorescent reactive oxygen sensors. Mol. Imaging Biol..

[7] Cheng T.C., Roffler S.R., Tzou S.C., Chuang K.H., Su Y.C., Chuang C.H., Leu Y.L. (2012). An activity-based near-infrared glucuronide trapping probe for imaging β-glucuronidase expression in deep tissues. J. Am. Chem. Soc..

[8] Keereweer S., Hutteman M., Kerrebijn J.D.F., van de Velde C.L.J.H., Vahrmeijer A., Lowik C.W.G.M. (2012). Translational optical imaging in diagnosis and treatment of cancer. Curr. Pharm. Biotechnol..

[9] Crane L.M.A., Themelis G., Arts H.J.G., Buddingh K.T., Brouwers A.H., Ntziachristos V., van der Zee A.G.J. (2011). Intraoperative near-infrared fluorescence imaging for sentinel lymph node detection in vulvar cancer: First clinical results. Gynecol. Oncol..

[10] Sevick-Muraca E.M. (2012). Translation of near-infrared fluorescence imaging technologies: Emerging clinical applications. Annu. Rev. Med..

[11] Troyan S.L., Frangioni J.V. (2009). The FLARE™ intraoperative near-infrared fluorescence imaging system: A first-in-human clinical trial in breast cancer sentinel lymph node mapping. Ann. Surg. Oncol..

[12] Te Velde E.A., Veerman T., Subramaniam V., Ruers T. (2010). The use of fluorescent dyes and probes in surgical oncology. Eur. J. Surg. Oncol..

[13] Kachala S.S., Servais E.L., Park B.J., Rusch V.W., Adusumilli P.S. (2010). Therapeutic sentinel lymph node imaging. Semin. Thorac. Cardiovasc. Surg..

[14] Aswathy R.G., Yoshida Y., Maekawa T., Kumar D.S. (2010). Near-infrared quantum dots for deep tissue imaging. Anal. Bioanal. Chem..

[15] Çamur M., Bulut M., Kandaz M., Güney O. (2009). Synthesis, characterization and fluorescence behavior of new fluorescent probe phthalocyanines bearing coumarin substituents. Polyhedron.

[16] Nesterova I.V., Erdem S.S., Pakhomov S., Hammer R.P., Soper S.A. (2009). Phthalocyanine dimerization-based molecular beacons using near-IR fluorescence. J. Am. Chem. Soc..

[17] Choi C.F., Tsang P.T., Huang J.D., Chan E.Y., Ko W.H., Fong W.P., Ng D.K. (2004). Synthesis and *in vitro* photodynamic activity of new hexadeca-carboxy phthalocyanines. Chem. Commun..

[18] Soares A.R., Tomé J.P., Neves M.G., Tomé A.C., Cavaleiro J.A., Torres T. (2009). Synthesis of water-soluble phthalocyanines bearing four or eight D-galactose units. Carbohydr. Res..

[19] Aggarwal A., Singh S., Zhang Y., Anthes M., Samaroo D., Gao R., Drain C.M. (2011). Synthesis and photophysics of an octathioglycosylated zinc (II) phthalocyanine. Tetrahedron Lett..

[20] Choi C.F., Huang J.D., Lo P.C., Fong W.P., Ng D.K. (2008). Glycosylated zinc (II) phthalocyanines as efficient photosensitisers for photodynamic therapy. Synthesis, photophysical properties and *in vitro* photodynamic activity. Org. Biomol. Chem..

[21] Kimani S.G., Shmigol T.A., Hammond S., Phillips J.B., Bruce J.I., MacRobert A.J., Golding J.P. (2013). Fully protected glycosylated Zinc (II) phthalocyanine shows high uptake and photodynamic cytotoxicity in MCF-7 cancer cells. Photochem. Photobiol..

[22] Lv F., Li Y., Cao B., Liu T. (2013). Galactose substituted zinc phthalocyanines as near infrared fluorescence probes for liver cancer imaging. J. Mater. Sci. Mater. Med..

[23] Lv F., He X., Lu L., Wu L., Liu T. (2012). Synthesis, properties and near-infrared imaging evaluation of glucose conjugated zinc phthalocyanines via Click reaction. J. Por. Phthal..

[24] Lv F., He X., Wu L., Liu T. (2013). Lactose substituted zinc phthalocyanine: A near infrared fluorescence imaging probe for liver cancer targeting. Bioorg. Med. Chem. Lett..

[25] Vuignier K., Schappler J., Veuthey J.L., Carrupt P.A., Martel S. (2010). Drug-protein binding: A critical review of analytical tools. Anal. Bioanal. Chem..

[26] Sułkowska A. (2002). Interaction of drugs with bovine and human serum albumin. J. Mol. Struct..

[27] Peters T. (1995). All about Albumin: Biochemistry, Genetics, and Medical Applications.

[28] Alarcón E., Edwards A.M., Garcia A.M., Muñoz M., Aspée A., Borsarelli C.D., Lissi E.A. (2009). Photophysics and photochemistry of zinc phthalocyanine/bovine serum albumin adducts. Photochem. Photobiol. Sci..

[29] Cheatum C.M. (2013). Drug-protein interactions: Mechanisms of potency. Nat. Chem..

[30] Bi S., Sun Y., Qiao C., Zhang H., Liu C. (2009). Binding of several anti-tumor drugs to bovine serum albumin: Fluorescence study. J. Lumin..

[31] Hein C.D., Liu X.M., Wang D. (2008). Click chemistry, a powerful tool for pharmaceutical. Pharm. Res..

[32] Tron G.C., Pirali T., Billington R.A., Canonico P.L., Sorba G., Genazzani AA. (2008). Click chemistry reactions in medicinal chemistry: Applications of the 1,3-dipolar cycloaddition between azides and alkynes. Med. Res. Rev..

[33] Lv F., Cao B., Cui Y., Liu T. (2012). Zinc phthalocyanine labelled polyethylene glycol: Preparation, characterization, interaction with bovine serum albumin and near infrared fluorescence imaging *in vivo*. Molecules.

[34] Joshi P., Chakraborty S., Dey S., Shanker V., Ansari Z.A., Singh S.P., Chakrabarti P. (2011). Binding of chloroquine-conjugated gold nanoparticles with bovine serum albumin. J. Colloid Interface Sci..

[35] Ross P.D., Subramanian S. (1981). Thermodynamics of protein association reactions: Forces contributing to stability. Biochemistry.

[36] Citrin D., Lee A.K., Scott T., Sproull M., Ménard C., Tofilon P.J., Kevin Camphausen K. (2004). *In vivo* tumor imaging in mice with near-infrared labeled endostatin. Mol. Cancer Ther..

[37] Meier R., Boddington S., Krug C., Acosta F.L., Thullier D., Henning T.D., Sutton E.J., Tavri S., Lotz J.C., Daldrup-Link H.E. (2008). Detection of postoperative granulation tissue with an ICG-enhanced integrated OI-/X-ray System. J. Transl. Med..

[38] Herbert B.J., Dorsey J.G. (1995). n-Octanol-water partition coefficient estimation by micellar electrokinetic capillary chromatography. Anal. Chem..

